# Effect of Layperson-Delivered, Empathy-Focused Program of Telephone Calls on Loneliness, Depression, and Anxiety Among Adults During the COVID-19 Pandemic: A Randomized Clinical Trial

**DOI:** 10.1001/jamapsychiatry.2021.0113

**Published:** 2021-02-23

**Authors:** Maninder K. Kahlon, Nazan Aksan, Rhonda Aubrey, Nicole Clark, Maria Cowley-Morillo, Elizabeth A. Jacobs, Rhonda Mundhenk, Katherine R. Sebastian, Steven Tomlinson

**Affiliations:** 1 Dell Medical School, The University of Texas at Austin; 2 Maine Medical Center Research Institute, Dell Medical School, The University of Texas at Austin; 3 Lone Star Circle of Care, Georgetown, Texas; 4 Seminary of the Southwest, Austin, Texas

## Abstract

**Importance:**

Loneliness is a risk factor for many clinical conditions, but there are few effective interventions deployable at scale.

**Objective:**

To determine whether a layperson-delivered, empathy-focused program of telephone calls could rapidly improve loneliness, depression, and anxiety in at-risk adults.

**Design, Setting, and Participants:**

From July 6 to September 24, 2020, we recruited and followed up 240 adults who were assigned to receive calls (intervention group) or no calls (control group) via block randomization. Loneliness, depression, and anxiety were measured using validated scales at enrollment and after 4 weeks. Intention-to-treat analyses were conducted. Meals on Wheels Central Texas (MOWCTX) clients received calls in their homes or wherever they might have been when the call was received. The study included MOWCTX clients who fit their service criteria, including being homebound and expressing a need for food. A total of 296 participants were screened, of whom 240 were randomized to intervention or control.

**Interventions:**

Sixteen callers, aged 17 to 23 years, were briefly trained in empathetic conversational techniques. Each called 6 to 9 participants over 4 weeks daily for the first 5 days, after which clients could choose to drop down to fewer calls but no less than 2 calls a week.

**Main Outcomes and Measures:**

Primary outcome was loneliness (3-item UCLA Loneliness Scale, range 3-9; and 6-item De Jong Giervald Loneliness [De Jong] Scale, range 0-6). Secondary outcomes were depression (Personal Health Questionnaire for Depression), anxiety (Generalized Anxiety Disorder scale), and self-rated health (Short Form Health Survey Questionnaire).

**Results:**

The 240 participants were aged 27 to 101 years, with 63% aged at least 65 years (n = 149 of 232), 56% living alone (n = 135 of 240), 79% women (n = 190 of 240), 39% Black or African American (n = 94 of 240), and 22% Hispanic or Latino (n = 52 of 240), and all reported at least 1 chronic condition. Of 240 participants enrolled, 13 were lost to follow-up in the intervention arm and 1 in the control arm. Postassessment differences between intervention and control after 4 weeks showed an improvement of 1.1 on the UCLA Loneliness Scale (95% CI, 0.5-1.7; *P* < .001; Cohen *d* of 0.48), and improvement of 0.32 on De Jong (95% CI, −0.20 to 0.81; *P* = .06; Cohen *d*, 0.17) for loneliness; an improvement of 1.5 on the Personal Health Questionnaire for Depression (95% CI, 0.22-2.7; *P* < .001; Cohen *d*, 0.31) for depression; and an improvement of 1.8 on the Generalized Anxiety Disorder scale (95% CI, 0.44 to 3.2; *P* < .001; Cohen *d*, 0.35) for anxiety. General physical health on the Short Form Health Questionnaire Survey showed no change, but mental health improved by 2.6 (95% CI, 0.81 to 4.4; *P* = .003; Cohen *d* of 0.46).

**Conclusions and Relevance:**

A layperson-delivered, empathy-oriented telephone call program reduced loneliness, depression, and anxiety compared with the control group and improved the general mental health of participants within 4 weeks. Future research can determine whether effects on depression and anxiety can be extended to maximize clinical relevance.

**Trial Registration:**

ClinicalTrials.gov Identifier: NCT04595708

## Key Points

**Question:**

Can a program of empathetic conversations delivered by laypeople via telephone reduce loneliness, depression, and anxiety in at-risk older adults?

**Findings:**

In this randomized clinical trial of 240 older adults receiving services through a Meals on Wheels organization, a 4-week empathy-oriented telephone program delivered by rapidly trained lay callers during the coronavirus disease 2019 pandemic improved loneliness, depression, anxiety, and general mental health.

**Meaning:**

In this study, loneliness, depression, and anxiety were rapidly reduced through layperson-delivered calls that focused on empathetic listening, suggesting a scalable approach to persistent mental health challenges of older adults.

## Teaser

This randomized clinical trial examines whether a layperson-delivered, empathy-focused program of telephone calls could rapidly improve loneliness, depression, and anxiety in at-risk adults.

## Introduction

Loneliness has been indicated as a risk factor for overall mortality and conditions from stroke to heart disease.^1^ It is associated with depression and anxiety, even if the direction and degree of causality is unclear.^2^ With the onset of coronavirus disease 2019 (COVID-19), there has been concern about the effect of increased isolation on loneliness and other mental health conditions.^3,4,5,6,7^ For older adults, those most socioeconomically vulnerable are likely to be at greatest risk.^8,9^

Few interventions have been shown to be effective,^1^ and the mental health workforce is already constrained. A systematic review of randomized interventions through 2010 found that structured, cognitive behavioral therapy (CBT)–based approaches were most effective but require trained counselors.^10^ In 2020,^11^ a video-conferenced behavioral activation intervention (a component of CBT) delivered by lay counselors over 5 weeks showed promising results.^11^

Comparison between studies is difficult because several tools are used to measure loneliness. Two prominent scales include the De Jong Giervald Loneliness Scale (De Jong) and the UCLA Loneliness Scale.^12^ The De Jong Scale is used in Europe, has been compared internationally,^13^ and may be useful for an elderly population.^10^ The 20-item UCLA Scale is frequently used in the United States and has a 3-item version for telephone administration.^14^ There are no established values to assess difference for clinically meaningful change.

In March 2020, we became aware of the challenges facing Meals on Wheels Central Texas (MOWCTX) clients because of reduced contact. In response, we designed a program that could be rapidly spun up and deployed. The telephone calls program involves laypeople engaging regularly, with empathetic intention, through telephone calls with participants. Empathy was functionally defined as prioritizing listening and eliciting conversation from the participant on topics of their choice. The protocol included an initial exposure to daily calls. Once exposed to the experience, participants chose the frequency of calls they prefer. Our goal was to test the program’s effectiveness in combating loneliness and other mental health conditions we expected may be worsening during COVID-19.

## Methods

This study was approved by University of Texas at Austin’s institutional review board on June 26, 2020. Participants provided verbal consent on the telephone. CONSORT reporting guidelines were followed.^15^ The formal trial protocols can be found in Supplement 1.

### Population

Participants were clients of MOWCTX. Staff introduced the study using a script and received permission to share contact information. Study personnel followed up via telephone to confirm interest and eligibility, obtain verbal consent to the research protocol, enroll, and collect baseline measurements. All MOWCTX clients were eligible except those with cognitive impairments, previously assessed through family report or via a case manager, or those in a hospice program.

### Intervention Program

Study personnel recruited callers through mailing lists and personal networks. Sixteen people from ages 17 to 23 years, including 14 college undergraduates, 1 person entering community college, and 1 graduate student, were recruited to deliver calls to participants (*callers*). Callers volunteered but were paid a stipend of $200 at the end of the program.

Callers were trained through a 1-hour videoconferenced session. The goal presented to callers was to learn from those they called by asking specific questions about topics raised by participants. No conversational prompts were provided nor training on CBT or its components. A short video was used to demonstrate techniques through role playing. Separately, callers received handouts and videotaped instructions on the logistics of the program (<1 hour).

Each caller supported a panel of 6 to 9 participants over 4 weeks. Calls were targeted to be less than 10 minutes; however, callers reported that calls could run longer. We did not limit time with the participant. Study personnel facilitated a 1-hour weekly, voluntary feedback session with callers.

The program was designed to maximize the participants’ perceived benefit. Calls were placed at the time of day participants requested. All participants were called 5 days during the first week. After this, participants chose the frequency of calls, with a minimum of 2 and maximum of 5 a week. Most (58%; n = 70 of 120) chose to continue to be called 5 times a week for the remaining 3 weeks; few (2%; n = 3 of 120) chose 4 a week, 17% (n = 20 of 120) chose 3 a week, and 22% (n = 27 of 120) chose 2 a week.

Callers used a Redcap system to track daily interactions, including whether a participant did not pick up, follow-up items for the next conversation, and any escalation-related issues. Calls were made through Amazon Connect and were not recorded.

The MOWCTX organization provided a list of escalation categories, including participant safety, food, or financial concerns. If a participant reported these concerns, the caller contacted MOWCTX staff to ensure the participant received a follow-up call. Thirty-four escalations were made during the study.

### Randomization and Blinding

Prior to randomization, participants were told they would either receive a program of calls for 4 weeks (intervention) or receive no calls until 4 weeks later for the follow-up and a $10 gift card (control). After consent and baseline measurements were completed, participants were randomized in blocks of 4 and 6 to intervention or control arms. A biostatistician did the randomization allocation, which was then uploaded to Redcap. In the intervention arm, participants were assigned to a caller’s panel once a sufficient number had consented so that each caller began with a full panel. Participants were called within 1 to 3 days after baseline collection. In the control arm, participants received no further contact until 4 weeks later, when they were called for follow-up assessment and subsequently sent the gift card.

A research associate, who was not involved in randomization, collected baseline and follow-up measures and was blinded to allocation arm, except for the final questions in the follow-up assessing satisfaction, which only displayed for intervention participants. Final assessments occurred 29 to 35 days after baseline.

### Measures

The primary outcome was loneliness, measured with the 6-item De Jong Scale (score range, 0-6)^16^ and the 3-item UCLA Loneliness Scale (score range, 3-9),^12^ higher numbers implying greater loneliness. Secondary outcomes included depression symptoms measured by the Personal Health Questionnaire for Depression (PHQ-8), anxiety symptoms measured by the Generalized Anxiety Disorder scale (GAD-7), social connection through the 6-item Lubben Social Network Scale (LSNS), and the 12-item general health questionnaire (Short Form Health Survey Questionnaire [SF-12], version 1.0, Mental and Physical Health components).^17^ We expected the physical scale of SF-12 and the LSNS not to be affected by this intervention; hence, they were included to help assess the specificity of the intervention effects. We measured demographic data through self-report based on investigator-defined categories, including age, sex, race/ethnicity, chronic conditions, medication use, marital status, and their degree of social interaction before and after COVID-19. Race/ethnicity was recorded to better prepare for replicability. Satisfaction was assessed at the end of the follow-up survey only for the intervention group (unblinded) through the question, “How satisfied were you with receiving the regular calls,” with a score of 1 to 5 (“very unsatisfied” to “very satisfied”).

### Statistical Analysis

The study was powered on the primary outcome measures with the assumption that the rank-order stability of the UCLA and De Jong instruments would be 0.6 from baseline to following intervention. Under those assumptions we targeted 125 participants in each arm to achieve 80% to 90% power to detect a small effect (*f* = 0.09 to *f* = 0.10) for differential change in the 2 arms, with α = .05. Not encountering predicted dropouts, we stopped recruitment at 120 in each arm that still had sufficient power for to detect an effect. To our knowledge, there are currently no prespecified clinically meaningful differences established for these measures.

We conducted linear and logistic mixed-effect regressions for all outcomes. Logistic mixed-effect regressions were run in addition to linear mixed-effect regressions when instruments had well-established clinical cutoffs for mild (5-9), moderate (10-19), or severe (>20) depression (PHQ-8)^18^ or anxiety (GAD-7)^19^ to gauge clinical significance. To accommodate the variability in the day of final assessment, the models were fit using the participant specific time that elapsed between baseline (set to day = 0) and the final assessment. The fixed-effects portion of the model only included the intercept, intervention group, days, and their interaction. The main effect of interest is the cross-level interaction of intervention group with days (ie, group by pre/post). We modeled person by time effects as nested in callers and assigned all participants in the control group to a single cluster to estimate and adjust the effect of clustering from shared callers. The models were fit with random effects of callers as well as participant intercept. We additionally tested for the random effect of days, but this term was not statistically significant for any of the outcomes. Although the random variance of callers was also not statistically significant for all outcomes except the mental health scale of SF-12, we retained it in the models to adjust for the small clustering effect of shared callers. To control for inflations of type I errors, Bonferroni corrections were applied separately to the primary outcomes (De Jong and UCLA loneliness; α = .025) and secondary outcomes (PHQ-8, GAD-7, and SF-12 mental health; α = .017). All analyses were conducted using Stata, version 16.1 (StataCorp), with full information maximum likelihood using an intention-to-treat framework.

## Results

### Participants

**Figure 1.**
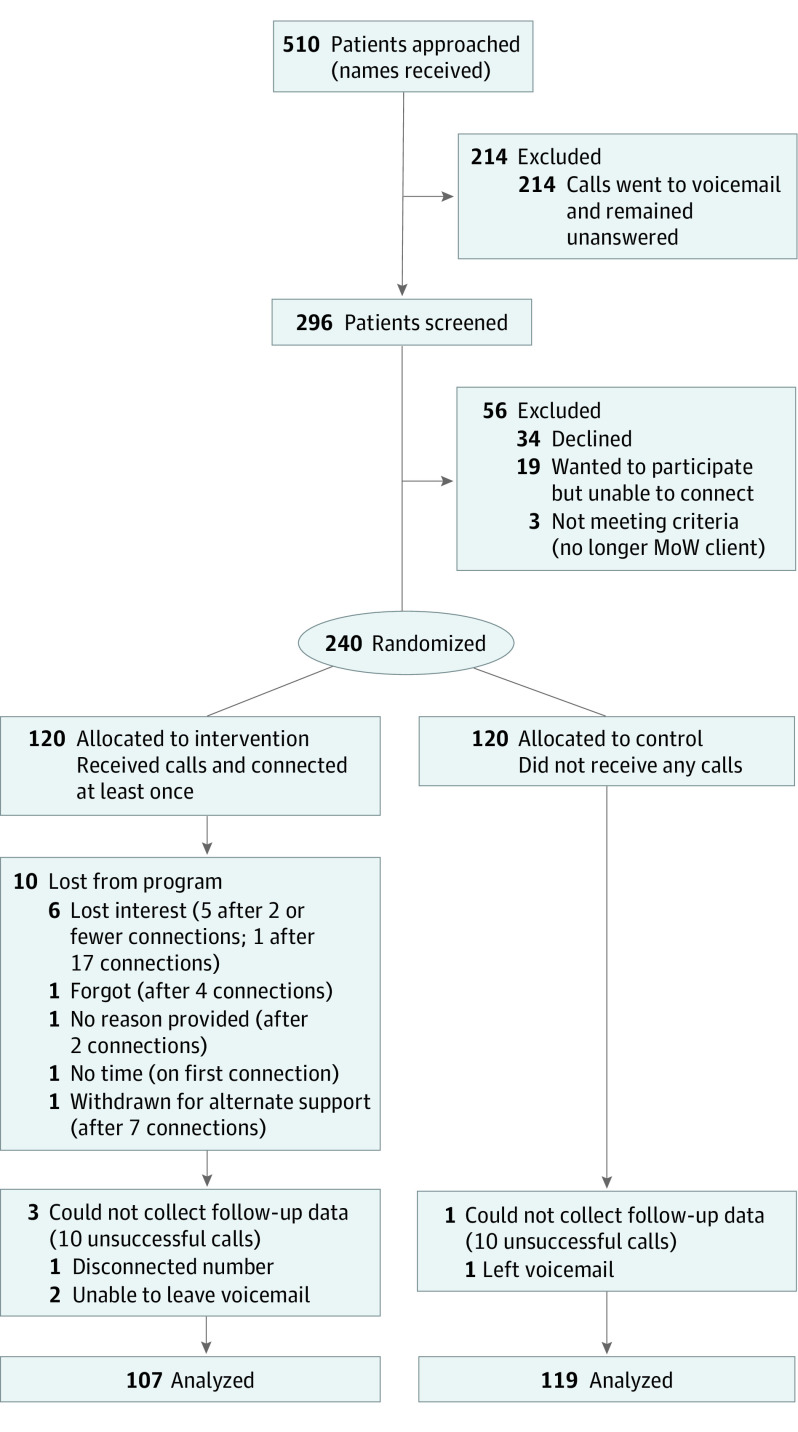
CONSORT Diagram: Flow of Participants Through the Trial

From July to August 2020, we received 510 referrals from MOWCTX, of whom 296 were successfully contacted. A total of 240 participants were enrolled and randomized into the study as described in the CONSORT diagram in Figure 1, with 120 in each arm. Of those in the intervention arm, 9 chose to drop out of the program, 7 on the 1st or 2nd, 1 on the 4th, and 1 on the 17th day of a connected call. One participant was removed from the program because of safety concerns that were escalated via MOWCTX to state services for support. After the program ended, 3 additional participants in the intervention arm and 1 in the control arm could not be contacted over 10 unsuccessful call days for follow-up data collection.

We compared the 13 individuals who were lost to follow-up in the intervention group with those who completed all of the assessments on baseline characteristics to assess patterns in dropout. Those who dropped out did not differ from those who completed all of the assessments on average age, chronic disease status, self-rated health, none of the primary or secondary outcome measures, or the distribution of categorical demographic characteristics such as sex and race/ethnicity distribution (White non-Hispanic vs other).

### Population

Table 1 shows the demographic characteristics of participants. More than one-third identified as African American, most were female, more than half were living alone, all participants in both arms had at least 1 chronic disease diagnosis, and self-rated health (range 0-4) was “good” on average in both groups. Only a minority reported being married; the rest were divorced, widowed, or single. These characteristics are typical of the MOWCTX client base. Most participants believed COVID-19 had changed their degree of social contact, although most still expressed that they received some visitors.

**Table 1.** Baseline Characteristics of the Sample in the Control and Intervention Arms

Characteristic	No. (%)	
	Control (n = 120)	Intervention (n = 120)
Age, mean (SD), y	68.7 (12.8)	69.4 (11.5)
Female	95 (79)	95 (79)
Ethnicity: Hispanic or Latino	26 (22)	26 (22)
Race		
American Indian	1 (1)	1 (1)
Asian	1 (1)	0
African American	49 (41)	45 (38)
White	42 (35)	51 (43)
Unreported	27 (23)	23 (19)
Chronic disease		
Diabetes	51 (42.5)	49 (41)
Heart disease	28 (23)	36 (30)
Kidney disease	6 (5)	10 (8)
High blood pressure	40 (33)	42 (35)
High cholesterol	8 (7)	6 (5)
Other	78 (65)	85 (71)
At least 1 medication	106 (90)	109 (92)
Living alone	68 (57)	67 (56)
Marital status		
Married	20 (17)	26 (22)
Single, divorced, widowed	100 (83)	94 (78)
Impact of COVID-19 on social contacts		
Visitors during COVID-19	72 (60)	68 (57)
Visitors pre–COVID-19	93 (78)	95 (79)
COVID-19 changed contact	80 (67.5)	79 (66)
Pre–COVID-19 outside activities	80 (67.5)	76 (63)
Self-rated health, mean (SD)	2.5 (.97)	2.5 (1.1)

Abbreviation: COVID-19, coronavirus disease 2019.

### Main Analysis

Participants in the intervention group improved from pre to post assessments of loneliness to a greater extent on the UCLA Scale than on the De Jong Scale; the latter did not achieve statistical significance (Table 2). Participants in the intervention group improved from a mean of 6.5 to 5.2 on the UCLA Scale and in the control group from 6.5 to 6.3 (group difference of 1.1; 95%CI, 0.5-1.1; *P* < .001; Cohen *d* of 0.48). Participants in the intervention group improved from a mean of 2.4 to 2.2 on the De Jong Scale, and in the control group did not change from a mean of 2.5 (group difference of 0.32; 95% CI, −0.20 to 0.81; *P* = .06).

**Table 2.** Loneliness, Mental and General Health, and Social Connections in Intervention and Control Arms at Baseline and After 4 Weeks of Intervention

Measurement	Time	Control	Intervention	Group difference at the postassessment stage		*P* value for group × days^b^	ICC (95% CI)^c^
		Mean (95% CI)	Mean (95% CI)	Mean (95% CI)	Cohen *d*^a^		
UCLA	Pre	6.5 (6.1 to 6.9)	6.5 (6.1 to 6.9)	1.1 (.50 to 1.7)	0.48	<.001	0.72 (0.62 to 0.80)
	Post	6.3 (5.9 to 6.8)	5.2 (4.8 to 5.6)				
De Jong	Pre	2.5 (2.1 to 2.8)	2.4 (2.1 to 2.8)	0.32 (−0.20 to 0.81)	0.17	.06	0.80 (0.75 to 0.84)
	Post	2.5 (2.1 to 2.8)	2.2 (1.8 to 2.5)				
LSNS	Pre	13.1 (11.9 to 14.3)	12.6 (11.3 to 13.8)	1.1 (−.67 to 3.0)	0.17	.37	0.80 (0.72 to 0.86)
	Post	13.0 (11.8 to 14.3)	11.9 (10.6 to 13.2)				
PHQ-8	Pre	6.2 (5.3 to 7.1)	6.3 (5.5 to 7.1)	1.5 (0.22 to 2.7)	0.31	<.001	0.78 (0.72 to 0.83)
	Post	6.3 (5.3 to 7.2)	4.8 (4.0 to 5.6)				
PHQ-8 ≥ 5, proportion (95% CI)^d^	Pre	0.58 (0.48 to 0.66)	0.59 (0.50 to 0.68)	0.09 (−0.03 to 0.23)	NA	.05	NA
	Post	0.54 (0.44 to 0.63)	0.44 (0.34 to 0.54)				
GAD-7	Pre	5.8 (4.8 to 6.9)	5.9 (4.9 to 6.9)	1.8 (0.44 to 3.2)	0.35	<.001	0.73 (0.66 to 0.78)
	Post	6.0 (4.9 to 7.0)	4.1 (3.2 to 5.0)				
GAD-7 ≥ 5, proportion (95% CI)^d^	Pre	0.49 (0.40 to 0.58)	0.50 (0.41 to 0.59)	0.14 (0.01 to 0.27)	NA	.02	NA
	Post	0.50 (0.40 to 0.59)	0.36 (0.27 to 0.45)				
SF-12 physical health	Pre	32.0 (29.9 to 34.1)	32.0 (29.8 to 33.2)	0.51 (−2.4 to 3.4)	0.05	.98	0.75 (0.69 to 0.80)
	Post	33.9 (31.8 to 36.0)	33.4 (31.4 to 35.3)				
SF-12 mental health^e^^,^^f^	Pre	44.3 (42.9 to 45.7)	42.5 (41.2 to 43.8)	2.6 (0.81 to 4.4)	0.46	.003	0.53 (0.44 to 0.63)
	Post	44.5 (43.3 to 45.7)	45.1 (43.8 to 46.3)				0.03 (0.01 to 0.57)

Abbreviations: De Jong, De Jong Giervald Loneliness Scale; GAD-7, Generalized Anxiety Disorder scale; ICC, intraclass correlation coefficient; LSNS, Lubben Social Network Scale; PHQ-8, Personal Health Questionnaire for Depression; SF-12, Short Form Health Survey Questionnaire; UCLA, UCLA Loneliness Scale.

^a^ Cohen *d* for the group difference at the postassessment stage.

^b^ *P* value for the interaction of group × days, the substantive effect of interest from mixed-effect regressions.

^c^ ICC and random variance approached 0 in all instances except for the mental health scale of the SF-12 for the caller’s cluster. In these instances, we did not include point estimates in the table.

^d^ The cells report the observed proportion meeting the clinical cutoff in each of the 4 cells, associated 95% CIs, and proportion differences. The *P* values and the ICCs are obtained from the mixed logistic regressions.

^e^ The direction of the interaction effect for SF-12 mental health scale concerned differences in within-group change rather than group differences at the postassessment stage. It is those differences that are presented in the group difference at the postassessment column of this table.

^f^ The ICC values given in the top concern participant clusters and the values of ICC below it concern caller clusters.

Depression and anxiety both improved in the intervention compared with the control group using continuous measures of these scales. Depression improved from a mean of 6.3 to 4.8 on the PHQ-8, and in the control arm, deteriorated from a mean of 6.2 to 6.3 (group difference of 1.5; 95% CI, 0.22-2.7; *P* < .001; Cohen *d* of 0.31). For participants in the intervention group, anxiety improved from a mean of 5.9 to 4.1 on the GAD-7, and in the control arm, deteriorated from a mean of 5.8 to 6.0 (group difference of 1.8; 95% CI, 0.44-3.2; *P* < .001; Cohen *d* of 0.35).

To evaluate the clinical relevance of the improvements in depression and anxiety, we examined whether the proportion of participants who were at least mildly symptomatic (anxious or depressed) at baseline (scores of ≥5 on both scales) decreased to asymptomatic status at the postassessment stage differentially for the intervention and control groups. Results were consistent with significant reductions in anxiety, with 50% of those with mild or greater anxiety (n = 60 of 120) in the intervention arm reducing to 36% (n = 38 of 107), while 49% of those with mild or greater anxiety (n = 59 of 120) in the control arm increased to 50% (n = 59 of 119) (group difference of 14%; 95% CI, 1%-27%; *P* = .02). Although there was a reduction in those with mild or greater depression in the intervention arm relative to the control arm, it was not statistically significant (group difference of 9%; 95% CI, −3% to 23%; *P* = .05). Finally, on the SF-12 mental health scale, intervention group participants’ scores improved from a mean of 42.5 to 45.1 and control participants’ scores improved from 44.3 to 44.5 (difference of 2.6; 95% CI, 0.81-4.4; *P* = .003; Cohen *d*, 0.46). Consistent with expectations, we did not see statistically significant changes in scores on the SF-12 Physical Health scale or in LSNS, which assesses more objective measures of social isolation. Finally, for participants in the intervention group, mean satisfaction was 4.52 (of a maximum of 5), with 65% of those assessed reporting as “very satisfied” and 88% reporting as “somewhat satisfied” or “very satisfied.”

**Figure 2.**
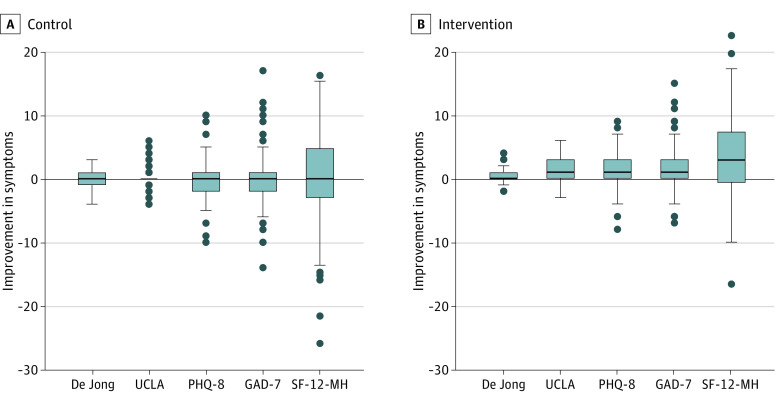
Box Plots of Changes in Loneliness, Depression, Anxiety, and General Mental Health Over 4 Weeks in the Intervention and Control Arms Box plot indicates median, interquartile range, and lower and upper adjacent values. De Jong indicates De Jong Giervald Loneliness Scale; GAD-7, Generalized Anxiety Disorder scale; PHQ-8, Personal Health Questionnaire for Depression; SF-12-MH, Short Form Health Survey Questionnaire Mental Health; UCLA, UCLA Loneliness Scale.

The effect sizes were generally small to moderate for those outcomes that showed a statistically significant difference in improvements between intervention and control groups as shown in box plots in Figure 2. Positive differences are consistent with improvement in a given outcome. Figure 2 depicts the improvements in the context of the wide range of baseline scores for all participants.

## Discussion

A 4-week, telephone-based, empathy-focused program delivered during the summer of 2020 reduced loneliness, depression, and anxiety in homebound, largely single, adults who require meals from a community-based provider.

Few prior programs have been shown to reduce loneliness through high-quality randomized trials. The studies that have shown moderate to larger reductions in loneliness implement some form of cognitive behavioral therapy.^10^ Choi et al^11^ showed improvements in loneliness of similar effect size to those we obtained when videoconferenced lay counselors implemented behavioral activation-focused sessions over 5 weeks with a similar population from Meals on Wheels organizations.^11^ Chiang et al^20^ showed a large improvement effect size (UCLA-20) for nursing home residents in Taiwan who were exposed to an 8-week reminiscence intervention. The reminiscence intervention focused on participants sharing experiences rather than a structured approach to maladaptive cognition. Both programs also significantly improved depression.

Our results are consistent with these prior studies and extend to effects on anxiety and general mental health. We did not screen for anxiety or depression, yet the program significantly reduced the proportion of participants who reported being at least mildly anxious at baseline.

The effect on loneliness varied in magnitude for the 2 instruments used to assess loneliness. The scales have slightly different item content and emphasize affective (UCLA) vs more cognitive (De Jong) approaches to understanding and measuring loneliness.^10^ The intervention presented here was designed to affect how people feel rather than how they think. This may explain the differential sensitivity of the 2 scales.

Compared with other intervention programs designed to reduce loneliness, our program required 2 hours of training for callers, no degree requirements, and no training on new tools for participants. The intervention was modeled as a continual support program, with higher frequency of contact in the first week dropping based on personal preference to lower frequency of contact. Although participants reported a high degree of satisfaction with the calls, we are unable to comment on whether the degree of empathy of callers or duration of conversations affected outcomes. However, caller characteristics likely had a minimal effect on reported outcomes because caller random variability was not significant in any of the models except for SF-12 mental health scale. However, all recruited callers were likely to want to serve this population, suggesting a potential factor in replicating these effects.

### Limitations

A major limitation of this study is that it is unclear whether benefits are sustained after 4 weeks. Two prior, successful, loneliness programs showed sustained effects 4 to 6 weeks after program delivery had ended.^11,20^ Future work should address whether improvements can be sustained, or enhanced, with a longer implementation period. Additionally, future research might explore the effect of this program when participants are screened for mental health conditions or stratified based on age. It may be particularly interesting to assess whether the program can play a protective role for those at risk of clinical anxiety or depression. Another limitation is that we cannot distinguish between the effect of being called vs the empathetic nature of the engagement. However, prior work has shown no impact of a weekly check-in call.^11^ We also observed higher dropouts in the intervention arm (n = 13) relative to control (n = 1), 7 occurring after only 2 connections, citing time and interest. Future program design might focus on minimizing early dropouts before participants have had a chance to experience program benefits. Finally, our study was not designed to uncover whether reductions in loneliness mediated improvements in mental health scores. Additionally, a strength and potential limitation of this study is that it was implemented during the COVID-19 pandemic.

## Conclusions

In this randomized clinical trial, a program of empathetic telephone calls tailored to participant preferences resulted in improvements in loneliness, depression, and anxiety over a 4-week period. The use of lay callers, deliberate but brief approach on training, and the use of ubiquitous telephones made the approach easily deployable and scalable.

## Notes

Supplement 1. Trial Protocol

Supplement 2. Data Sharing Statement
