# Supplementary material for: Effect of Layperson-Delivered, Empathy-Focused Program of Telephone Calls on Loneliness, Depression, and Anxiety Among Adults During the COVID-19 Pandemic: A Randomized Clinical Trial
Source: JAMA Psychiatry. 2021 Feb 23;78(6):1–7. doi: 10.1001/jamapsychiatry.2021.0113 (PMC7903319; doi:10.1001/jamapsychiatry.2021.0113)
Supplement: Supplement 1. — Trial Protocol [file jamapsychiatry-e210113-s001.pdf]

The University of Texas at Austin
Institutional Review Board
Standard Submission Form

STUDY INFORMATION

1

For studies following a multi-center or sponsor protocol, please use this guidance to assist in your completion of this form.

For questions regarding definitions, policies, or terms referenced below see the policies and procedures manual.

2

Study Title and Number from IRBaccess
Telephone Calls for Health for Homebound Older Adults
2020-05-0009

3

Principal Investigator			
Name	Position	UT EID	E-mail Address
Maninder Kahlon	Assoc Professor, Pop Health; Vice Dean for Health Ecosystem	Mk34328	mkahlon@austin.utexas.edu
If principal investigator is a student, describe how the PI is qualified/trained to conduct this study.			
Click or tap here to enter text.			

4

Faculty Sponsor *(required if the PI is a student)*			
Name	Position	UT EID	E-mail Address
First Last	Title	XXX##	jdoe@utexas.edu
Describe how faculty sponsor will oversee the conduct of the study.			
Click or tap here to enter text.			

5

Primary Point of Contact (if different from PI)			
Name	Position	UT EID	E-mail Address
Rhonda Aubrey	Senior Business Health Director	Rca959	Rhonda.aubrey@austin.utexas.edu

6

Additional Research Staff	
☒	Research staff other than the principal investigator will conduct human subject research.

If additional personnel will be engaged in conducting research human subject research, complete and upload the Research Personnel Form

Engaged in human subject research is defined as contact or interaction with research

participants through recruitment, informed consent process, data collection, analysis of or access to identifiable research data.

7

Purpose and Rationale for Conducting Research
Hypothesis
We hypothesize that a telephonically delivered engagement intervention with possible referral for additional services, reduces loneliness in a cohort of home-bound older adults that qualify for Meals on Wheels (MOW) services, relative to a control group that receives 2 survey collection calls, and a $10 gift card, but does not receive the intervention calls.
Study Background
Nearly one out of three older Americans now lives alone with a high risk of experiencing social isolation and loneliness. A review of the literature indicates that social isolation and loneliness significantly contribute to poor health outcomes and ultimately mortality. Individuals who engage in unhealthy habits such as smoking, or physical inactivity or do not maintain a healthy weight, but have close social ties, live longer than those who engage in healthier lifestyles but lack these important social connections.<br><br>Loneliness has been associated with depressive symptoms that remain significant across socioeconomic statuses. One study demonstrated that belonging to groups and maintaining social connections can be a preventive factor in developing depression and attenuating depression symptoms in individuals who are diagnosed with depression.<br><br>Older adults have been identified as belonging to a particularly high-risk group during the COVID 19 pandemic. As a result, reducing the exposure of seniors to others has been a strategy to protect them from contracting the virus. This strategy, however, has resulted in seniors experiencing greater social isolation as compared to the pre-COVID period. MOW normally delivered a hot meal to their senior clients 5 days each week, primarily using a volunteer distribution system. Since COVID, MOW has stopped their production and delivery of hot meals and instead shifted to delivering shelf-stable food left in a box at the door once every two weeks. For safety purposes, they have eliminated the human connection. The organization is concerned about the overall well-being of their clients and are interested in identifying solutions that could address the greater social isolation and possible loneliness as a result of their change in meal distribution.<br><br>Studies that look at the relationships between clinical providers and their patients have identified that patients value affective concerns, or empathy, as much as, if not more than their perception of their clinician's technical competence. A systematic review of studies that looked at empathy in communications with patients, identified empathy as having unquestionable importance in the health outcomes of patients.<br><br>1. Holt-Lunstad, J., Smith, T., Baker, M., Harris, T., Stephenson, D. Loneliness and Social

Isolation as Risk Factors for Mortality: A Meta-Analytic Review. *Perspectives on Psychological Science.* 2015, Vol. 10(2) 227–237.
2. Anderson, G., Thayer, C. . Loneliness and Social Connections: A National Survey of Adults 45 and Older. Washington, DC: AARP Research, September 2018. https://doi.org/10.26419/res.00246.001.
3. Cacioppo, J., Hughes, M., Waite, L., Hawkley, L., Thisted, R. Loneliness as a Specific Risk Factor for Depressive Symptoms: Cross-Sectional and Longitudinal Analyses. *Psychology and Aging* 2006, Vol. 21, No. 1, 140–151.
4. Steger, M., Kashdan, T. Depression and Everyday Social Activity, Belonging, and Well-Being. *Couns Psychol.* 2009 April ; 56(2): 289–300. doi:10.1037/a0015416.
5. Halpern J. What is clinical empathy? *J Gen Intern Med*. 2003;18(8):670-674.
6. Derksen, F., Bensing, J., Lagro-Janssen, A. Effectiveness of empathy in general practice: a systematic review. *Br J Gen Pract* 2013; DOI: 10.3399/bjgp13X660814

Design and Methodology

Design:

Design: Randomized controlled trial of effect of 4 weeks of regular check-in calls, up to 5 per week based on participant's choice and 2 survey collection calls and possible referral of other services, versus no daily check-in calls, but the two survey collection calls and gift card receipt, on self-reported loneliness measures for current Meals on Wheels participants (MOW).

Recruitment: MOW staff will contact current clients and, using a script, introduce the study and seek permission to share the client's contact information with the research team for those clients who express possible interest in participating in the study. Clients with cognitive impairment or in hospice care will not be eligible for participation. MOW staff will provide the research team with the client's name, telephone number, and address in batches of 25 names. The research associate will outreach to the MOW clients to consent the participants. MOW will continue to outreach to identify interested participants until 250 clients have been consented and enrolled into the study by the research team.

Randomization: Names of participants who are consented and enrolled in the study will be shared with the research manager who will randomize the participants into either the control or the intervention arm using Redcap randomization functionality. To ensure balance over time, treatment assignment will be block randomized with blocks in random sizes of either four or six, wherein the research associate will be blinded to block and blinded to the next treatment assignment in the randomization list. The statistical team will prepare the randomization model. Participants in the intervention will be added to the caller's call schedule to begin the calls on the next business day.

The intervention arm will include 125 MOW clients to receive a call of between 5 - 10 minutes in length each by a consistent caller, five times a week, Monday through Friday for 4 consecutive weeks to check in on them. After the first week of calls, subjects in the intervention

arm will be asked if the frequency of calls is acceptable or if they would like to reduce the call frequency, potentially to a minimum of twice per week.

1. Each caller has between 20-25 participants in a daily panel. 4-6 callers are in the process of recruitment.
2. Each call is 5 - 10 minutes in length.
3. The intervention will last 4 weeks for each MOW client.
4. Callers will use a telephone system that identifies their calling organization as Dell Med in caller id. Telephone number called, date of call, and length of call will be captured automatically.

Callers: Brief guidance and training will be provided for daily conversation to maximize time spent speaking by the recipient but otherwise the calls are unscripted other than specific prompts to ask about the recipient's general sense of well-being. "How are you doing today?"

Issue Sharing: We will utilize MOW's current process, used by their volunteer food deliverers, to share issues identified during the intervention calls that might result in additional service offered by MOW. Should an unexpected urgent issue be identified by the caller that is related to the health and safety of the participant, the PI will be contacted. The PI will reach out to the MOW case managers to ensure the health and safety of the study participant. Additionally, participants may give permission to share their survey tool results with MOW, in which case, MOW will address concerning survey scores.

<u>Control Arm</u>: 125 MOW clients will be randomized to a control group that will not receive the intervention calls. The control group will receive calls from a member of the research team at the beginning of the study to collect baseline survey data and at the post-4-week period to collect post-study survey data. Participants in the control group will receive a $10 HEB or Visa gift card upon completion of the study.

<u>Timeline</u>: 1) Baseline data collection. 2) Week 1-4 – intervention is delivered. 3) Post intervention data collection. We expect the intervention to begin 1-2 days after baseline. And we expect the post intervention data collection to occur 1-2 days after the end of the last call.

Other goals of study:
In addition to the primary aim of the study, we will also be collecting information to inform future studies including a current IRB approved program scheduled to begin after the summer (whenever COVID guidelines permit) on the impact of medically-tailored meals and social connection on Hemoglobin A1C and other health measures for diabetics in the MoW program. Additional goals include:

a. Impact of intervention on the validated tools we will use for the future study – Depression/PHQ-8, Anxiety GAD-7
b. Comparison of the primary loneliness tool used in this study, the 6-item De Jong Gierveld Loneliness Scale, with the results from the short-form UCLA loneliness tool that is currently

planned for our longer study.

Sub-study exploration goals:
A second, but related area of exploration within this study will allow us to begin understanding the impact of caller empathy on outcomes, and self-reported alignment of empathy level compared to perceived empathy as reported by call recipient. This embedded sub-study requires that the callers, members of the research team delivering the intervention in the primary study, are consented and measurements collected from them in their roles as study participants within the sub-study.

c. Perception of caller's approach and impact (CARE measure, completed by intervention arm of main study)
d. Assessment by each caller of their empathetic ability via the Toronto Empathy Questionnaire.

Data Analysis

Primary outcome: Loneliness as measured by the 6-item De Jong Gierveld Loneliness Scale; secondary outcomes include depression and anxiety. All three outcome measures yield continuously distributed summary scores with interval scale properties; and all three outcomes are measured both prior to and after the intervention period. This design permits us to examine whether the phone-based loneliness intervention differentially improves the loneliness scores over that of the control group. Mixed linear models/ hierarchical linear models will be adopted to model pre to post change in loneliness at level-1; we will test whether the pre to post changes in loneliness differs as a function of treatment assignment at level-2. These analyses will control and test for differences in the magnitude of the treatment effect across the 6 callers at level-2. Participant ratings pertinent to perception of the caller's empathy/impact measured by CARE as part of the sub-study will also be included at level-2. A similar analytic approach will be adopted to test if the phone-based loneliness intervention also affects depression and anxiety ratings from pre to post-intervention. The rank-order stability of loneliness, depression, and anxiety scores tend to be moderate to large; and moderate to high correlations affect statistical power positively. A sample size of 125 participants in each group can detect a small effect size for the interaction of group with pre to post-difference (f= .09 to f= .10) with 80% to 90% power respectively using an alpha of .05 assuming a correlation of .60 between the pre and post measurements. The sample size of 125 participants in each group has been established to adequately power the study to provide information on the effect of the intervention to inform future planned studies. Mixed linear models will permit the estimation of these models when there is missing data while accounting for greater similarity in therapeutic effects in subsets of participants due to shared callers who deliver the intervention. The exploratory analyses pertinent to perception of the impact/approach of the caller by the participant will be incorporated into these primary analyses and will inform whether the perception of effectiveness predicts the magnitude of change from pre to post intervention.

8 Funding and Regulatory Oversight

Check all agencies that fund or hold regulatory oversight over the research activities.

If study activities are regulated by the FDA, check FDA here. The FDA regulates any experiment that involves a test article and one or more human subjects, and that either must meet the requirements for prior submission to the FDA or the results of which are intended to be later submitted to, or held for inspection by, the FDA as part of an application for a research or marketing permit..

☐	Food and Drug Administration (FDA) Regulated				
☐	NIH	☐	Department of Defense (DoD)<br>*Complete Supplemental IRB Application - DoD*	☐	Dept. of Education (DoEd)
☐	Dept. of Energy (DOE)	☐	Department of Justice DOJ/NIJ	☐	Environmental Protection Agency (EPA)
☐	Bureau of Prisons				
☐	Other Federal Agencies: Click here to enter text.				
☐	Industry/Private Sponsor: Click or tap here to enter text.				
	UT Funding Account Number: Click or tap here to enter text.				
☒	Other External Funding: Episcopal Health Foundation				
OSP: Click or tap here to enter text.					

9

PROCEDURES

10

Study Elements

Check any that apply to your study. This is not meant as a comprehensive record of your entire study.

A full description of all study procedures should be provided in the procedures section below or the applicable supplement form.

☐	Bio-specimen<br>*Complete Supplemental IRB Application – Biospecimens*	☐	Biometrics	☐	Registry or repository<br>*Complete Supplemental IRB Application - Repository*
☐	Focus Group	☐	Genetic Analysis	☐	Genomic Data Sharing
☐	International research<br>*Complete Supplemental IRB Application - International*	☒	Interview/ Survey	☐	MRI
☐	PHI<br>*Complete Supplemental IRB Application - PHI*	☐	Observation	☐	Record Review (Prospective)
☐	Record Review	☐	Screening Procedures	☐	Sensors (Externally Placed)

	(Retrospective)				
☐	Sensors (Inserted)	☐	Video/Audio Recording	☐	X-Ray
Interventions					
☐	Drug/Biologic<br>*Complete Supplemental IRB Application - Drugs*	☐	Device<br>*Complete Supplemental IRB Application - Device*	☐	Behavioral
Additional Oversight					
☐	Biohazards, Recombinant DNA, or Gene Transfer<br>*Upload IBC approval letter*	☐	Human embryonic, human induced pluripotent, or human totipotent stem cells; or human gametes or embryos	☐	Radiation exposure without direct clinical benefit<br>*Upload radiation safety approval*
Additional Questions:					
☒	This study involves one or more human subjects who are prospectively assigned to one or more interventions (which may include placebo or other control) to evaluate the effects of those interventions on health-related biomedical or behavioral outcomes.				

11

Procedures
Describe all study procedures, including a step-by-step outline of what participants will be asked to do or how data will be used. Be sure to describe all of the following in detail, as applicable:<br>*a) All study procedures, in sequential order*<br>*b) All research measures/tests that will be used (state if questions or measures are standardized or published)*<br>*c) Secondary data or specimens that will be obtained, how they will be collected, and how they will be used*<br>*d) Where each activity will take place, the duration of each, and who will perform each activity*<br>*e) Include time commitment of participants*<br>*f) Mark all optional procedures as [OPTIONAL]*
a) **Study Procedures in sequential order.**<br><br>**i. Recruitment**<br>MOW staff will call current clients drawing from their information management system. Clients with documented cognitive impairment or in a hospice program will not be called. MOW staff will use a script to introduce the telephone intervention study and seek permission to share the client's contact information. For clients who assent, MOW will forward client names and telephone numbers preference on to the research team in an Excel spreadsheet using Box, a secure data sharing option, in batches of 25. MOW will continue placing interest calls to clients until such time as they are notified by the research team that the 250 study participants have been consented and enrolled.

Procedures

ii. Consenting

A member of the research team will call the MOW clients to explain the program details. They will begin the consenting process by reading the consent form to the MOW client. Potential participant will be given time to ask any questions about the research and the time commitments involved. A verbal consent, including date of consent and name of research team member receiving consent will be captured in the Redcap system which we will be using for all data collection. Participants will be verbally provided with the contact information for the research team and and the Institutional Review Board for any concerns and questions they may have.

iii. **Baseline measurement**.

After consent has taken place, a member of the research team will obtain baseline measurements. The call to obtain baseline measurements will take approximately 30 minutes.

iv. **Randomization**.

Study participants will be randomized after baseline measurement are completed using Redcap randomization functionality and as designed by the statistical team, as a block randomization.

Participants randomized into the control group will be told they are in the second arm of the program, that they will receive a call after 4 weeks (for measurement), and to expect to receive an HEB or Visa gift card after that. In the intervening 4 weeks, they will not receive any additional contact by research team until the post-4-week measurement data is collected.

v. Program Intervention

For those randomized to the intervention arm, the following program elements will be implemented:

The client name and telephone number, will be placed on the caller's call schedule to begin calling on the next business day.

During the first call, the caller will introduce themselves and remind the participant about the program elements. The content of the call will include a question asking how the participant is doing, otherwise the call will be unscripted. The caller will be able to document the call content in the phone system as a reminder of topics shared by the participant for future call continuity. Should an issue that requires sharing with MOW be identified, as pre-determined by MOW case management staff, the caller will forward the

Procedures

issue details to MOW on the same day. Resolution of the issue will be determined by MOW and could potentially result in additional services provided by MOW. Should an unexpected urgent issue be identified by the caller that is related to the health and safety of the participant, the PI will be contacted via email and text. The PI will reach out to the MOW case managers to ensure the health and safety of the study participant.

During the first call of the week for weeks 2-4, the participant will be asked if the frequency of the calls is acceptable, providing the participant with agency and choice. Requests to reduce the frequency of calls to a minimum of two calls per week will be used to adjust the call schedule for the week.

Each call will last approximately 5 - 10 minutes.

Additionally, the intervention arm will receive survey phone calls by the research associate at the beginning and end of the 4 week study period.

b) **All Research Measures**
All measures, with the exception of the CARE survey, are collected via a telephone call at baseline and the standardized surveys at the post-4-week period. The CARE survey will only be asked of the participants in the intervention arm during the post-4 week measurement period. No protected health information will be obtained and no aggregate data will be collected as part of this study.
Primary:

**Outcome Measures**	**Collection Tool**	**Frequency**
Demographics (Name, Age, Sex, Race, Language, Phone number, Address)	Redcap Survey	Baseline
Household Status	Redcap Survey	Baseline
Self-reported medical conditions with date of diagnosis	Redcap Survey	Baseline and post-intervention
Intervention Satisfaction	Redcap Survey	Post-intervention (for intervention arm only)
Loneliness	De Jong Gierveld Loneliness Scale (6-item) (standardized, published)	Baseline and

Procedures

		post-intervention
Loneliness/Isolation	UCLA Short Scale for Measuring Loneliness in Large Surveys<br><br>(Standardized, published)	Baseline and<br><br>post-intervention
Depression	PHQ-8<br><br>(Standardized, published)	Baseline and<br><br>post-intervention
Anxiety	GAD7<br><br>(Standardized, published)	Baseline and<br><br>post-intervention
Social Isolation & Connection	Lubben Social Network Scale<br><br>(Standardized, published)	Baseline and<br><br>post-intervention
Consultation & Relational Therapy	CARE Measure<br><br>(Standardized, published)	Post-intervention (for intervention arm only)
Quality of Life	Health Status and Quality of Life SF12 (Standardized, published)	Baseline and<br><br>post-intervention

Sub-Study Exploration on Importance of Volunteer Caller Empathy:

**Outcome Measures**	**Collection Tool**	**Frequency**
Demographics (Name, Age, Sex, Race, Language)	Redcap Survey	Baseline
Empathy	Toronto Empathy Questionnaire<br><br>(Standardized, published)	Baseline and<br><br>post-intervention

c) **Secondary data**
No secondary data.

d) **Where each activity will take place, duration of each, and who will do each**
All activities of the study will take place telephonically.
Roles: Research Manager (not blinded to study arm assignment), Research Associate (blinded to group assignment as this role will be collecting the baseline and post intervention surveys), Program Associate/Caller (not-blinded, only delivers calls to the

Procedures

intervention arm).

i. **Recruitment –** approximately 5 minutes per call, by Research Associate
ii. **Consenting –** 30 minutes per participant, conducted by Research Associate
iii. **Baseline measurement –** 30 minutes per participant, conducted by Research Associate
iv. **Intervention calls –** 5 days/week M-F for 4 weeks taking 5-10 minutes per call, conducted by Program Associate/Caller.
v. **Post-intervention measurement -**30 minutes per participant, conducted by Research Associate.

e) **Time commitment of participants.**

i. **Intervention arm**

Cumulative number of hours for the intervention calls is approximately 3 and 1/3 hours (20 calls lasting approximately 10 minutes each).

Cumulative number of hours for the baseline (which will include the consent process and any questions and 4-week post intervention measurement collection for the participant is approximately 1.5 hours.

ii. **Control arm**

Cumulative number of hours for the baseline and 4-week post intervention measurement collection for the participant is approximately 1.5 hours.

Sub-study exploration

a) Sub-Study Procedures in sequential order.

i. **Consent-**

As members of the research team in the role of callers for the primary study, each Program Associate/Caller will learn of the goals of the sub-study and their role as participants for the sub-study exploration. The consent form will be shared with them via email. These members have already been identified as volunteer callers for the main study. Participation in the sub-study is completely optional and will not impact their role as a member of the research team in the primary study, nor will it impact their relationship with UT moving forward. They will be given the opportunity to ask questions about the sub-study via telephone or email. The consent will be obtained via electronic signature by a member of the research team.

ii. **Measurement collection**-

Procedures
Program Associates/Callers for the primary study will be asked to complete the Toronto Empathy Questionnaire prior to the empathy/engagement training or placing any calls for the primary study intervention and after completion of the 4 weeks of calls<br><br>**iii. Empathy/Engagement training**<br>A limited, approximately one hour session, will be provided to the Program Associates/Callers that reviews the role of empathy in building connections with others and provides suggestions on tools that could be used to effectively engage with the intervention arm participants of the main study. This limited training will be standardized across the course of the trial.<br>.<br>**b) All research measures**<br>Toronto Empathy Questionnaire (standardized, published)<br><br>**c) Secondary data**<br>None<br><br>**d) Where each activity will take place, duration of each, and who will do each**<br>All activities will take place virtually, either via email or telephonically.<br>Consenting – 10 minutes per participant, conducted by member of the research team<br>Measurement Collection – 5 minutes per participant per survey, collected by a member of the research team<br><br>**e) Time commitment of participants**<br>Program Associates/Caller participants **–** 20 minutes

12

Alternatives to Participation in this Study
Current MOW clients would continue to receive normal MOW services.

13

LOCATIONS

14

Study Locations

Identify the sites where study activities will occur under the direction of UT Investigators.					
☒	UT Austin	☐	UT Health Austin	☐	Dell Seton Medical Center<br>*Upload S.A.T. submission receipt*
☐	Dell Children's Medical Center<br>*Upload S.A.T. submission receipt*	☐	K-12 schools/district	☐	Day care center
☐	Seton Medical Center Austin<br>*Upload S.A.T. submission receipt*	☐	CommunityCare		

15

External Locations	
Include any non-UT site where UT or non-UT personnel will conduct consent, data collection, intervention, or analysis of identifiable data under the direction of the UT principal investigator.<br><br>*If UT Austin will serve as the reviewing IRB for a multi-site study (study involves collaboration with sites or individuals external to UT Austin who are engaged in human subjects research), contact RSC to verify the UT IRB will serve as the reviewing IRB.*<br><br>*Once verified, each relying site must complete the IRB Reliance Form.*	
Site Name	IRB Oversight Plan
.No External locations	Select IRB Oversight.
Additional Questions	
Will UT act as a central coordinating site?	No
Describe procedures to communicate SAEs, UPs, and modifications to external sites.	Click or tap here to enter text.

16

17

SUBJECT POPULATION

18

Protected Subject Populations					
Select all populations specifically studied under this research.					
☐	Active military personnel	☐	Children	☐	Decisionally impaired adults
☐	Emancipated minors	☐	Fetuses	☐	Individuals with limited

					English proficiency
☐	Neonates	☐	Pregnant women	☐	Prisoners<br>*Complete [Supplemental IRB Application - Repository]*
☒	UT Students				

Research Participant Information				
Describe the research population.				
Participant Groups:	• **Primary Study, Intervention and Control groups, current MoW clients**<br>• **Sub-study, 7 Program Associates/Caller participants**			
Age range	18	To	99	
Gender	Any			
Inclusion criteria	Current MOW client. For sub-study, research team members in the role of caller for primary study			
Exclusion criteria	Cognitive impairment or in hospice program. For sub study, have a close relationship with the primary investigator that might be perceived to impact their course as a UT student			
Population info	Primary study: Homebound older adults that are clients of MOW.<br>Sub-study: former or current UT students			

Total Sample Size	
Total number of participants for all participant groups	N = 250 for sub-study N = 7
Sample size rationale	A sample size of 125 participants in each group can detect a small effect size for the interaction of group with pre to post-difference (f= .09 to f= .10) with 80% to 90% power respectively using an alpha of .05 assuming a correlation of .60 between the pre and post measurements. The sample size of 125 participants in each group has been established to adequately power the study to provide information on the effect of the intervention to inform future planned studies.

SCREENING & RECRUITMENT

Identification and Screening	
☐	This study involves obtaining information or biospecimens for the purpose of screening, recruiting or determining eligibility of prospective subjects prior to informed consent by either:<br>1. Oral or written communication with the prospective subject or LAR<br>2. By accessing records containing identifiable private information or stored identifiable biospecimens
	Describe the identification and/or screening procedures:
	Click or tap here to enter text.

Recruitment					
Select all recruitment methods utilized for this research and describe the recruitment process. Upload copies of recruitment materials/scripts to IRBaccess.					
☐	E-Mail	☐	Flyer	☐	In-Person
☐	Letter	☐	Social Media	☐	Research Pool
☒	Telephone/Text	☐	Snowball sampling	☐	Web-posting
☐	Word of Mouth	☐	Other: Click or tap here to enter text.		
Describe the recruitment process including where recruitment will take place.					
MOW staff will call current clients drawing from their information management system. Clients with documented cognitive impairment or in a hospice program will not be called. MOW staff will use a script to introduce the telephone intervention study and seek permission to share the client's contact information. For clients who assent, MOW will forward client names and telephone numbers on to the research team in an Excel spreadsheet using Box, a secure data sharing option, in batches of 25. MOW will continue placing interest calls to clients until such time as they are notified by the research team that the 250 study participants have been consented and enrolled.					

OBTAINING INFORMED CONSENT

Consent Overview
Select all applicable.

See IRB Policies and Procedures Section 6 for a description of informed consent.<br>*See IRB Policies and Procedures Section 12.4 for a description of assent/parent permission.*			
☐	Obtaining Written Consent<br>*Complete the Consent and Assent Processes section below*	☒	Requesting Waiver of Documentation of Informed Consent<br>*Complete the Consent and Assent Processes and the Waiver of Documentation of Consent sections below*
☐	Requesting Waiver of Informed Consent<br>*Complete Waiver or Alteration of Informed Consent section below*	☐	Requesting Alteration of the Required Elements of Informed Consent<br>*Complete Waiver or Alteration of Informed Consent section below*
☐	Obtaining Child Assent<br>*Complete the Consent and Assent Processes section below*	☒	Obtaining Short Form Consent<br>*Complete the Consent and Assent Processes section below*

29

Consent and Assent Processes
Provide a detailed description of the consent process including who will obtain consent, where, and when consent will occur in such a manner that participants have sufficient time for adequate consideration.
Due to the high-risk nature of this senior, homebound population, our intention is to conduct all aspects of the study via virtual, telephonic means. For the consent process, a member of the research team will call interested MOW clients on the phone, as identified by MOW, and introduce themselves. The informed consent form contents will be read to the possible participants. Possible participants will be given the opportunity to ask any questions about the study that they have and offered time for consideration prior to obtaining verbal consent. Verbal consent will be obtained via telephonic engagement and documented by the research associate.<br><br>**Sub-study exploration**<br>UT student volunteers recruited to make calls for the primary study will be invited to participate in the sub-study via phone call and will receive a copy of the consent via email. A senior member of the research team will review the consent document with the volunteer researcher/sub-study participant and answer questions about the study. Sub-study participants, who have already been selected and on-boarded for the primary study, will be informed of the voluntary nature of the sub-study and that the decision to participate will have no impact on their relationship with UT Austin or their grades if they are a UT student. If the volunteer researcher agrees to participate, the consent form will be signed via DocuSign.
Upload consent forms, script, or letter to IRBaccess.

Waiver of Documentation of Consent	
To approve a waiver of documentation of informed consent, one of the following criteria below must be justified by the researcher.<br><br>*Only complete the section below if requesting a waiver of documentation of informed consent.*	
☐ Waiver Option 1<br><br>A) The only record linking the subject and the research would be the consent document<br><br>B) The principal risk would be potential harm resulting from a breach of confidentiality.<br><br>C) Each subject will be asked whether the subject wants documentation linking the subject with the research, and the subject's wishes will govern.	*Click or tap here to enter text.*<br><br>*Upload consent forms with and without signature lines.*<br><br>*Include this choice in the informed consent form.*<br><br>*Articulate the destruction protocol for signed consent forms in the privacy and confidentiality section.*
☒ Waiver Option 2<br><br>A) This study is minimal risk.<br><br>B) Written consent would not be required outside of the research context	All activities within the study will be conducted telephonically. This study is minimal risk. Consenting for a phone call intervention and survey collections would not require written consent outside of the research context.<br><br>*Upload consent form to IRBaccess.*

☐ Waiver Option 3<br><br>A) The subjects or legally authorized representatives are members of a distinct cultural group or community in which signing forms is not the norm<br><br>B) the research presents no more than minimal risk of harm to subjects.<br><br>C) There is an appropriate alternative mechanism for documenting that informed consent was obtained.	.
	Upload consent form to IRBaccess

Waiver or Alteration of Informed Consent	
To approve a waiver of informed consent, all of the following criteria must be justified by the research. Provide a protocol specific justification for each.<br><br>*Only complete the section below if requesting a waiver of informed consent or alteration of informed consent.*	
The research involves no more than minimal risk to the subjects.	Click or tap here to enter text.
The waiver or alteration will not adversely affect the rights and welfare of the subjects.	Click or tap here to enter text.

The research could not practicably be carried out without the waiver or alteration (it is impracticable to perform the research if obtaining informed consent is required and not just impracticable to obtain consent).		
If the research involves using identifiable private information or identifiable biospecimens, the research could not practicably be carried out without using such information or biospecimens in an identifiable format.	Click or tap here to enter text.	
Whenever appropriate, the subjects will be provided with additional pertinent information after participation.	☐	Additional pertinent information would not be appropriate (*e.g.*, no deception).
	☐	Additional pertinent information is appropriate.
		Research that requires alteration of informed consent on the grounds that deception is necessary must complete the deception section below.

Deception	
See IRB Policies and Procedures Section 15 for a description of deception.	
Describe the nature of deception	*Click or tap here to enter text.*
Why is deception required?	*Click or tap here to enter text.*
Describe debriefing procedures	*Click or tap here to enter text.*

☐	Research participants will have the opportunity to withdrawal their data during the debriefing.
Upload debriefing form to IRBaccess.	

34

Consent Translation

☐	The study population will likely include participants whose limited English speaking status requires translation of the consent form.	
The IRB recommends having English versions of consents approved prior to translation.<br>When available, upload translated documents to IRBaccess.<br><br>See IRB Policies and Procedures Section 6.4.1 for a description of translation procedures.		
☐	The consent documents will be translated by a certified translator.	
☐	A non-certified translator will translate the consent documents.	
	Describe the translator's qualifications	
	Click or tap here to enter text.	
	☐	Documents will be translated, and the research team will attest that the translation is accurate and appropriate.
Upload translated documents and attestation (if required) to IRB Access.		

RISKS AND BENEFITS

Benefits		
Compensation for time and effort is not considered a benefit.		
Benefits to Society		Describe scientific and societal benefit.
		**Primary study:** Increasing numbers of older adults are living alone in the community. Given potential deaths of spouses, family and friends, retirement, and age-related physical changes, the aging adult becomes high-risk for social isolation and loneliness, both of which can contribute to poor health outcomes. Findings from this study will help to inform potential intervention development to address this growing health issue.<br>**Sub-study exploration**: Growing evidence suggests that the degree of empathy perceived within a relationship contributes to desired, successful outcomes of the relationship. By exploring the self-reported empathy levels of the caller, the perceived empathy levels by the participant, and the achieved outcomes of the primary study intervention, it could help inform future programs about the characteristics most important to identify in program personnel initiating the social connection, and the importance of such to the program's effectiveness
Direct Benefit	☐	No potential for direct benefits to participants
	☒	Describe potential for direct benefits to participants.
		Participants in the intervention arm of the primary study could

		benefit from the intervention by reducing their level of loneliness, depression, anxiety, and social isolation.<br><br>There is no direct benefit to the sub-study participants.

38

Risks	
☐	Greater than Minimal Risk Study<br>*Complete the Data Safety and Monitoring Plan section below.*
Research related risks only pertain to risks associated with procedures required by the study; do not include risks of any procedures that the participant would undergo if not participating in the study.	
Describe the risk(s) associated with the research.	
The measures are minimal risk.	
Describe the risk mitigation plan	
Although there are minimal risks to participating in this part of the study, there is potential for discomfort sharing personal information about themselves. We have indicated in the consent form that the participants do not have to answer any questions that make them feel uncomfortable and they can stop the interview and the intervention calls at any time. There is also the risk of loss of confidentiality. We will follow HIPAA security standards in data transition and storage to minimize this risk.	

39

Data Safety and Monitoring Boards (DSMBs) and Plans (DSMPs)	
☐	This study will have a DSMB.
	Describe the DSMB including frequency of meetings, members, data reviewed, and stopping points.
	Click or tap here to enter text.
☐	The study will have a DSMP.
	Describe the DSMP, including what data or responses are monitored, when data is reviewed, and what actions are taken to react to a safety concern.

40

Required Consent Disclosures		
Child and Elder Abuse		
Texas law requires that anyone report suspected child/elder abuse or neglect.		
Is it likely investigators could discover information that would require mandatory reporting by the investigators or staff?	☐	Yes, it is likely.<br>*Include mandated reporting language in applicable informed consent document(s).*
	☒	No, it is not likely.

Incidental Findings	
Incidental findings include: genetic markers, concerning test results, disease, suicidal thoughts, unexpected paternity, engaging in illegal activities.	
☒	It is possible that investigators could discover incidental findings or other information about a participant's previously unknown condition.
	If so, state methods for addressing and reporting incidental findings
	Issues of concern that would be identified by Meals on Wheels volunteers during their typical process of meal delivery may be identified during calls with the Meals on Wheels clients, such as missing meal deliveries or prescription access needs. We will use the same process to share issues that has been developed by Meals on Wheels for their volunteer meal deliverers
	Include incidental report information as applicable in the informed consent document(s).

Early Withdrawal
List the criteria for withdrawing individual participants from the study (*e.g.*, safety or toxicity concerns, emotional distress, inability to comply with the protocol, or requirements from study sponsor).
Participants may withdraw from the study at any point.
Describe any necessary procedures for ensuring the safety of a participant who has withdrawn early.
None
Describe any pre-specified criteria for stopping or changing the study protocol due to safety concerns.
None
If any of the above are applicable, include this information in your consent form.

PRIVACY AND CONFIDENTIALITY

Privacy
Describe how you will protect the identity and privacy of study participants during each phase of research. Privacy focuses on the individual participants rather than data. In this section, researchers should focus on issues such as where research activities take place and how participant involvement is protected from non-participants.
Include information regarding privacy during identification, recruitment, screening, the consent process, the conduct of the study, and dissemination of data.
Identification (via Meals on Wheel staff), screening, recruitment, and the intervention will take place over the phone which will reduce the risk of loss of privacy. All documents, including consent forms, will be kept on secure servers within Dell Medical. Any hard copies of documentation will be stored in locked filing cabinets in controlled-access buildings within Dell Medical

Confidentiality and Data Security Plan

Describe how you will protect the confidentiality of data or address confidentiality concerns.		
☐	Identifiers will be coded to protect confidentiality.	Describe how data is coded and where identifiers are stored.
☒	Identifiable data will be destroyed.	Describe destruction plan and timeline
		Identifiable data will be deleted from data and lists at the end of the study.
☐	Identifiable data will not be destroyed.	Provide rationale for retaining identifiable data indefinitely.
		Click or tap here to enter text
Describe how you will store and secure your data (including length, location, and medium of storage):		
All data will be stored in the Dell Medical School REDCap database. This secure, HIPAA compliant database can only be accessed with password and dual authentication		

46

Data Access					
☒	Study team members	☐	Collaborators	☐	Data coordinating center
☐	Sponsor	☐	Future sharing with other researchers	☒	Other: Meals on Wheels Central Texas
Describe Data Sharing (If Applicable)					
Identifiable research data will be shared outside of the research team, with Meals on Wheels, unless the participants give explicit denial to share identifiable data with Meals on Wheels post-study. Data sharing with Meals on Wheels would be for the purpose of internal use only to better support the care provided to the Meals on Wheels client. This would include referral of services suggested by a participant's survey scores. A member of the research team will ask specifically if the participants do not want to share data with MOW. Aggregate data without identifiers will be shared with healthcare payers, and may be presented in public forums					

47

Certificate of Confidentiality		
See IRB Policies and Procedures Section 4.11.5 for a description of a Certificates of Confidentiality.		
☒	The study does not require a Certificate of Confidentiality.	
☐	The study requires a Certificate of Confidentiality.	
	☐	NIH has issued a Certificate of Confidentiality for this study.
	☐	A Certificate of Confidentiality has not been obtained, but there are plans to apply for one.

48

COMPENSATION AND COSTS

49

Compensation					
☒	Subjects receive compensation.				
☐	Subject will not receive compensation.				
Total amount of compensation		$10<br>Control group participants will receive a $10 HEB or Visa gift card upon completion of the post-intervention measurement collection. This will be emailed or mailed to the participant based on their preferred method.			
Proration schedule		Click or tap here to enter text.			
When do subjects receive compensation?		Click or tap here to enter text.			
Select the form(s) of compensation					
☐	Cash	☐	Check	☒	Gift Card $10 HEB or Visa gift card
☐	Course Credit	☐	ClinCard	☐	Other: Click or tap here to enter text.
☒	Compensation amount and type reasonable for this population for the activities requested of them.				
	We use standards from similar prior approved and completed work				

50

Costs			
Select all categories of costs for which participants or their insurance companies will be responsible.			
☒	Participants will have no costs associated with this study		
☐	Standard of care procedures contributing to study data	☐	Research procedures not associated with standard of care
☐	Administration of drugs / devices	☐	Study drugs or devices
☐	Transportation and parking		
☐	Other: Click or tap here to enter text.		

51

52

REQUIRED DOCUMENTS

53

Additional Supporting Documents	
☒	Principal Investigator CV - Required

☐	Faculty Sponsor CV – Required for student PIs
☒	Recruitment Materials
☒	Consent, Parental Permission, and Assent Forms
☒	Measures and Instruments
☐	Sponsor Protocol
☐	Investigator Brochure
☒	Personnel Form
☐	IDE/IND Verification
☒	Supplemental Forms

UT Austin IRB Approved
Protocol Number:
Approved:

Title of the Project: Telephone Calls for Health for Homebound Older Adults
Principal Investigator: Maninder Kahlon, PhD, Vice Dean, Health Ecosystem Dell Medical School; Assoc Professor, Population Health
Study Sponsor: Episcopal Health Foundation

*** This form will be read out loud to potential participants and verbal consent will be collected. ***

Meals on Wheels Client Consent to Participate in Research

Invitation to be Part of a Research Study

You are invited to be part of a research study. This consent form will help you in choosing whether or not to participate in the study. Feel free to ask if anything is not clear in this consent document.

What is the study about and why are we doing it?

The purpose of the study is to see if receiving phone calls from volunteers helps you feel less lonely and reduce feelings of depression or anxiety. We think this research program has the potential to help older adults or other adults who are homebound, like yourself, feel more connected to other people, especially during the COVID19 pandemic.

What will happen if you take part in this study?

If you agree to take part in this study, you will be asked to:

- You will be asked to complete a series of survey questions about your feelings of loneliness, connection, anxiety and depression. These questions will take approximately 30 minutes to complete over the phone.
- Next, you will be randomly assigned to be in one of two groups. Which group you end up in is entirely up to chance, much like a coin toss.
- If you are randomly assigned to the intervention group, you will receive a 5- 10 minute phone call every Monday – Friday for four weeks (20 days) with an opportunity to reduce the number of calls you receive each week after the first week.
- If you are randomly assigned to the control group, you will not receive additional calls from UT, Dell Med research volunteers on a daily basis.
- Finally, regardless which group you are assigned to, the UT research team will call you and again ask you a series of survey questions about your feelings of loneliness, connection, anxiety, and depression. These surveys will be completed over the phone today and again at the end of the program, in about 4 weeks. It will take about 30 minutes to complete the surveys during each phone call.

How long will this study take and how many people will be in the study?

Participation in this study will take place over a period of four weeks and include 250 participants, 125 in the intervention group and 125 in the control group. Participants in the intervention group will receive calls every weekday lasting approximately 10 minutes, for four weeks, for a total of roughly 200 minutes (just under 3.5 hours). Both intervention and control

groups will be asked survey questions at the start and end of the study (approximately 4 weeks after the study start surveys) that take up to 30 minutes each for a total of one hour.

Intervention group total time commitment = 4.5 hours
Control group total time commitment = 1.0 hour

What risks and discomforts might you experience from being in this study?

There are some risks you might experience from being in this study. There is a risk that some questions might make you feel uncomfortable. You do not have to answer any question that makes you feel uncomfortable. There is a potential for accidental release of confidential information. Procedures are in place to minimize this risk.

How could you benefit from this study?

You may receive phone calls every weekday for four weeks from a member of the UT research team. Callers will check-in on you, ask you about your day, and how you are feeling. These calls may help make your day more pleasant, enhance your overall wellbeing, or assist you in connecting to Meals on Wheels emergency help if you need it.

What data will we collect from you?

As part of this study we will collect information about your wellbeing using surveys that measure things like depression, loneliness, anxiety, and social connection. We will also record the phone calls made by the volunteers if you are placed in the group receiving daily phone calls.

How will we protect your information?

We will protect your information by using a coded participant number and a secure database. Only research staff have access to this database. UT Austin research team members and Meals on Wheels staff will have access to your identifying information, such as your name and phone number. In addition, Meals on Wheels will obtain access to individual survey information collected by the UT Austin researchers to provide better service in the future. If you do not want the data shared with Meals on Wheels, please let us know and we will not share your data.

The recordings of the daily phone calls will be stored on a secure server with access allowed only to limited the research operations manager. Access will require identification and password.

Information about you may be given to the following organizations:

- Representatives of UT Austin and the UT Austin Institutional Review Board

We plan to publish the results of this study. To protect your privacy, we will not include any information that could directly identify you.

Under certain situations, we may break confidentiality. If during the study, we learn about child or elder abuse or neglect, or that someone is a clear, serious, and direct harm to self or others, we may report the information to the appropriate authorities, including the police, the Texas Department of Family and Protective Services, and/or an emergency medical facility.

A description of this study will be available on http://www.ClinicalTrials.gov as required by U.S. Law. This web site will not include information that can identify you. At most, the web site will include a summary of the results. You can search this web site at any time.

What will happen to the information we collect about you after the study is over?

Your name and other information that can directly identify you will be deleted from the research data collected as part of the project.

How will we compensate you for being part of the study?

Participants in the UT Dell Medical intervention group will not receive payment for participation in the study.

Participants in the control group will receive a $10 HEB or Visa gift card for their participation upon completion of the study.

Your Participation in this Study is Voluntary

It is totally up to you to decide to be in this research study. Participating in this study is voluntary. Your decision to participate will not affect your relationship with The University of Texas at Austin or Meals on Wheels. You will not lose any benefits or rights you already had if you decide not to participate. Even if you decide to be part of the study now, you may change your mind and stop at any time. You do not have to answer any questions you do not want to answer.

Contact Information for the Study Team and Questions about the Research

If you have any questions about this research, you may contact:

Maninder “Mini” Kahlon
Phone: 512-495-5017
Email:mkahlon@austin.utexas.edu
Address: ATTN: Mini Kahlon
1501 Red River St.
Austin, TX 78701
Mail Code: Z0100

Or

Maria Cowley-Morillo
Phone: 512-584-1376
Email: maria.cowleymorillo@austin.utexas.edu

Contact Information for Questions about Your Rights as a Research Participant

If you have questions about your rights as a research participant, or wish to obtain information, ask questions, or discuss any concerns about this study with someone other than the researcher(s), please contact the following:

The University of Texas at Austin
Institutional Review Board
Phone: 512-232-1543

Email: irb@austin.utexas.edu

Please reference study number 2020-05-0009.
