# Supplementary material for: Effect of Layperson-Delivered, Empathy-Focused Program of Telephone Calls on Loneliness, Depression, and Anxiety Among Adults During the COVID-19 Pandemic: A Randomized Clinical Trial
Source: JAMA Psychiatry. 2021 Feb 23;78(6):1–7. doi: 10.1001/jamapsychiatry.2021.0113 (PMC7903319; doi:10.1001/jamapsychiatry.2021.0113)
Supplement: Supplement 2. — Data Sharing Statement [file jamapsychiatry-e210113-s002.pdf]

## Data Sharing Statement

### Data

**Data available:** Yes
**Data types:** Data dictionary
**How to access data:** Request sent to Nicole Clark
nicole.clark@austin.utexas.edu
**When available:** With publication

### Supporting Documents

**Document types:** None

### Additional Information

**Who can access the data:** Researchers whose proposed use of the data has been approved.
**Types of analyses:** For qualified researchers, to better understand what we did and to suggest additional analyses.
**Mechanisms of data availability:** The data dictionary can be made available freely. We also look forward to sharing data, but at this time have further analyses we are doing ourselves, and based on researcher interest, would collaborate on analyses with a signed research agreement.
